# Shorter Height is Associated with Diabetes in Women but not in Men: Nationally Representative Evidence from Namibia

**DOI:** 10.1002/oby.22394

**Published:** 2019-02-25

**Authors:** Viola Koncz, Pascal Geldsetzer, Jennifer Manne‐Goehler, Amanda S. Wendt, Felix Teufel, S.V. Subramanian, Till Bärnighausen, Jan‐Walter De Neve

**Affiliations:** ^1^ Institute of Global Health, Medical Faculty and University Hospital Heidelberg University Heidelberg Germany; ^2^ IBE Institute for Medical Information Processing, Biometry and Epidemiology Ludwig Maximilian University of Munich Munich Germany; ^3^ Department of Global Health and Population Harvard T.H. Chan School of Public Health Boston Massachusetts USA; ^4^ Department of Medicine Beth Israel Deaconess Medical Center, Harvard Medical School Boston Massachusetts USA; ^5^ Department of Social and Behavioral Sciences Harvard T.H. Chan School of Public Health Boston Massachusetts USA; ^6^ Center for Population and Development Studies Harvard T.H. Chan School of Public Health Boston Massachusetts USA; ^7^ Africa Health Research Institute KwaZulu‐Natal South Africa

## Abstract

**Objective:**

This study aimed to test the hypothesis that attained adult height, as an indicator of childhood nutrition, is associated with diabetes in adulthood in Namibia, a country where stunting is highly prevalent.

**Methods:**

Data from 1,898 women and 1,343 men aged 35 to 64 years included in the Namibia Demographic and Health Survey in 2013 were analyzed. Multiple logistic regression models were used to calculate odds ratios (ORs) and 95% CIs of having diabetes in relation to height. The following three models were considered: Model 1 included only height, Model 2 included height as well as demographic and socioeconomic variables, and Model 3 included body mass index in addition to the covariates from Model 2.

**Results:**

Overall crude diabetes prevalence was 6.1% (95% CI: 5.0‐7.2). Being taller was inversely related with diabetes in women but not in men. In Model 3, a 1‐cm increase in women’s height was associated with 4% lower odds of having diabetes (OR, 0.96; 95% CI: 0.94‐0.99; *P *= 0.023).

**Conclusions:**

Height is associated with a large reduction in diabetes in women but not in men in Namibia. Interventions that allow women to reach their full growth potential may help prevent the growing diabetes burden in the region.

## Introduction

Diabetes prevalence is rising rapidly in low‐ and middle‐income countries 1. Africa in particular is expected to have the largest proportional increase of diabetes among adults worldwide by the year 2040 2. Major risk factors include obesity, rapid urbanization, physical inactivity, aging, nutrition transitions, and broader socioeconomic changes 3. However, growing evidence has suggested that conditions during pregnancy or in childhood play an important role in the development of diabetes later in life 4. This early programming of diseases has led to the “developmental origins of health and disease” concept (also known as the “thrifty phenotype hypothesis”) 5, which links aspects of an early unfavorable environment to risks of chronic disease later in life. Indeed, animal models have suggested that intrauterine malnutrition can alter glucose metabolism via several structural and functional pathways, including impaired pancreatic insulin secretion, decreased insulin sensitivity of muscle cells, increased fat storage in the adipose tissue, and altered appetite regulation in the brain 6. A key assumption of the disease programming concept is that its effects are most pronounced when there is a mismatch between early nutritional deprivation and later nutritional affluence, leading to an abandonment of an individual’s growth trajectory 7, 8. Obesity in particular would play a critical role in the association between intrauterine restriction, early childhood growth, and adult diabetes 9. In sub‐Saharan Africa, obesity is a growing concern, while low birth weight (< 2,500 g) and early childhood undernutrition remain high, further emphasizing the relevance of the developmental origins theory in low‐resource and transitional countries 8.

Stunting, defined as height or length for age below −2 standard deviations (SDs) of the World Health Organization (WHO) Child Growth Standards median 10, is the most prevalent form of undernutrition in childhood globally 11 and has a negative influence on the physical and mental development of affected children later in life 12. In addition to possible intrauterine growth restriction, growth faltering in children typically begins at about 6 months of age with transition to foods that are often inadequate in quantity and quality 13, and growth continues to decline until about 24 months 14. A small window of opportunity for catch‐up growth would exist from conception through the first 2 years of life 15. Persisting stunting beyond the first 1,000 days, however, is strongly associated with shorter attained adult height 4, 12, creating an intergenerational cycle of stunted mothers with stunted offspring 16.

To date, most of the existing studies on the programming of disease concept have focused on body weight, especially birth weight 17, rather than on body height. The current focus on body weight might have harmful repercussions, as relative weight gain after the age of 2 years may have adverse effects on later cardiovascular risk factors 18, whereas linear growth in early life could lead to substantial gains in human capital in adults without further increasing the burden of noncommunicable diseases 18. Only a handful of studies, however, have assessed the association between attained adult height and diabetes. Height appears inversely related with diabetes in Iran 19, Israel 20, Denmark 21, The Netherlands 22, and the United States 23, whereas in Portugal, no significant association was found 24. A cohort study from Germany and a meta‐analysis from nine studies in primarily developed settings found a divergent association between women and men 25, 26. In a five‐country cohort study, including Brazil, Guatemala, India, Philippines, and South Africa, conditional height (defined as present height accounting for previous height) was unrelated to dysglycemia 18. Taken together, the limited available evidence on the association between adult height and diabetes has appeared inconsistent, and most studies have been conducted in relatively affluent settings. Although the programming of disease concept is not restricted to certain populations, its effects may be more pronounced in populations with a high prevalence of early childhood undernutrition, such as in Namibia.

Namibia has one of the highest levels of early childhood stunting globally. The prevalence of stunting in children under 5 years was 24% in 2013, though this represents a decline from 29% in 2006 27. Children in rural areas are particularly prone to stunting possibly because of limited access to formal education, frequent exposure to diarrhea, relative poverty, and shorter duration of breastfeeding 28. Contrary to the WHO and United Nations International Children's Emergency Fund (UNICEF) recommendation that children under 6 months of age be exclusively breastfed, only 49% of infants under age 6 months are exclusively breastfed in Namibia, and only 41% of children aged 6 to 23 months are fed the minimum number of times 27. Moreover, Namibia has the highest prevalence of obesity among adults in sub‐Saharan Africa (25.5% and 8.0% among women and men, respectively) 29. The coexistence of malnutrition among both children and their parents suggests a potentially large role for developmental origins of acquiring diabetes later in life. The age‐standardized prevalence of diabetes is estimated to have increased from 3.8% to 7.5% in women and from 2.6% to 7.3% in men between 1980 and 2014 30.

To address the existing gap in the literature, this study provides evidence on the relationship between attained adult height, as an indicator of childhood nutritional status, and diabetes in Namibia, a low‐resource setting where stunting is highly prevalent. Attained adult height is a useful marker of cumulative net nutrition during major growth periods and can be used as a proxy for early life living standards 31, 32. We hypothesized, based on the programming of disease concept, that individuals who likely experienced malnutrition in early life in the Namibian context, without sufficient catch‐up growth, may have higher odds of having diabetes compared with individuals with normal height development. To our knowledge, this study is the first to investigate the association between attained adult height and diabetes in sub‐Saharan Africa using a nationally representative survey design.

## Methods

### Data source and study population

Data was extracted from the 2013 Namibia Demographic and Health Survey (DHS), a nationally representative population‐based household survey. DHS surveys are designed to collect data on fertility, family planning, and maternal and child health to assist countries and researchers in assessing changes in population, health, and nutrition 27. The 2013 Namibia DHS was implemented by the Ministry of Health and Social Services together with the Namibia Statistics Agency and the National Institute of Pathology. The sampling frame used for this survey was the preliminary frame of the Namibia Population and Housing Census conducted in 2011. For the sampling approach, each of the 13 administrative regions was divided in small enumeration areas covering the entire country. In a two‐stage process, 20 households were randomly selected in every urban and rural cluster. In addition to demographic, socioeconomic, and health data, the 2013 Namibia DHS measured the prevalence of high fasting plasma glucose (FPG) and collected anthropometric measurements of weight and height. A total of 9,849 households were successfully interviewed, yielding a household response rate of 97%. Anthropometric characteristics and FPG were investigated in a subsample of women and men aged 35 to 64 years. Of the 2,584 women and 2,163 men eligible for the FPG test, 74% of women and 63% of men had their plasma glucose measured 27. In our analysis, we included only participants with full information on our exposure, outcome, and covariates, yielding a total study population of 1,898 women (73% of eligible women) and 1,343 men (62% of eligible men) aged 35 to 64 years.

### Outcome

Our outcome was diabetes using WHO cutoff points. FPG values ≥ 7.0 mmol/L (126 mg/dL) were defined as diabetes 33. Plasma glucose was measured in capillary whole blood after an overnight fast. Although the WHO recommends venous plasma glucose as the standard method for measuring glucose concentrations in blood, capillary sampling is widely used, particularly in low‐resource settings. Fasting values for venous and capillary plasma glucose are considered to be identical 33. All respondents included in our study confirmed that they had fasted for at least 8 hours prior to measurement. In addition to a blood test, participants were asked whether they were taking medication for diabetes (“Are you currently receiving prescribed medication such as insulin for your high blood glucose or diabetes?”). Individuals reporting use of drugs for diabetes were classified as having diabetes, irrespective of their biomarker values. Our outcome for diabetes was treated as a dichotomous variable (diabetes/no diabetes).

### Exposure

Our key exposure was attained adult height as a continuous variable in centimeters. Adult height was measured once in a standing position using a Shorr height board (Weight and Measure, LLC, Maryland, USA). Measurements were performed by trained staff following a detailed field manual.

### Covariates

We included sex, age (continuous), residency (urban, rural), educational attainment (level attained at the time of the survey), and household wealth quintile as covariates to control for potential confounding in our analysis, in addition to body mass index (BMI) as a potential mediator. The household wealth index is a composite measure of a household’s cumulative living standard. It is calculated using data on a household’s ownership of selected assets and is typically based on 25 to 50 survey questions, such as materials used for housing construction and types of water access and sanitation facilities. The wealth index is generated based on principal components analysis (as per standard DHS methodology) and places individual households on a continuous scale of relative wealth. In the DHS, interviewed households were separated into five wealth quintiles (five being the wealthiest) 27. BMI was grouped into four categories, thin (BMI < 18.5 kg/m^2^), normal (BMI 18.5‐24.9 kg/m^2^), overweight (BMI 25‐29.9 kg/m^2^), and obesity (BMI ≥ 30 kg/m^2^), in accordance with the WHO classification 34. Data on waist circumference or ethnicity were not available in the DHS.

### Statistical analyses

The main objective of our analysis was to investigate the relationship between attained adult height and diabetes, controlling for potential confounders. Three different multiple logistic regression models were fitted. The models considered were Model 1, including only height to examine the unadjusted effect of height; Model 2, including height along with demographic and socioeconomic variables (age, sex, residency, education, wealth); and Model 3, including BMI along with the covariates of Model 2. We tested our assumption of a linear relationship between attained adult height and diabetes by adding higher‐order polynomial terms of height into the models, but none of the associations became significant at the 0.05 level in the pooled data of women and men with the exception of Model 1. We therefore kept height in the models as a linear term. All regression analyses were conducted in the pooled study sample, as well as stratified by sex to determine sex‐specific associations. Odds ratios (ORs) with 95% CIs for correlates of diabetes were estimated. Sample weights were used as provided by the DHS for all descriptive statistics. We clustered standard errors at the enumeration area level to take into account spatial correlation between respondents.

### Sensitivity analyses

We conducted a wide range of supplementary analyses to generate additional confidence in the robustness of our findings. First, we used an alternative specification of our exposure. We transformed height into a categorical variable based on quartiles, in which Q1 represents the shortest and Q4 the tallest height category. Second, we included BMI as a continuous variable in our model to fully adjust for BMI instead of the BMI categories thin, normal, overweight, and obesity. Third, we used alternative specifications for age and BMI to model the nonlinear relationship of age and BMI with diabetes risk. We included quadratic and cubic terms in age as well as BMI in our models. Fourth, we restricted our sample to workers and included occupation as a covariate in our models to further account for lifestyle differences. We used the occupation categories as provided by the DHS, which are based on the International Standard Classification of Occupations. Fifth, we excluded pregnant women (*n* = 35) from our analytical sample because pregnancy may affect blood glucose. Sixth, we modeled our outcome with a log link function in Poisson regression models. Seventh, we graphically assessed the relationship between the proportion of children under 5 years of age who were stunted and mean attained adult height by region in Namibia.

Lastly, we conducted a placebo test. We restricted our analytical sample to married couples and ran four additional models in which we regressed the following: (1) wife’s diabetes status on wife’s height, (2) wife’s diabetes status on husband’s height, (3) husband’s diabetes status on wife’s height, and (4) husband’s diabetes status on husband’s height. We hypothesized that if adult height could be interpreted as a true (causal) exposure of diabetes, the coefficients from Models 2 and 3 would likely be substantially smaller relative to the coefficients obtained from Models 1 and 4, respectively. Conversely, if the coefficients from, for instance, Models 1 and 2 would be similar in effect size, a possible causal interpretation of Model 1 would appear problematic and suggest possible residual socioeconomic confounding or assortative mating 35. Stata (version 15.0; StataCorp, College Station, Texas) was used for all statistical analyses.

### Ethical clearance

This study was considered exempt from full review by the Harvard T.H. Chan School of Public Health Institutional Review Board, as the analysis was based on an anonymous public use data set with no identifiable information on the survey participants.

## Results

The mean age of our study sample was 46.9 (SD 8.2) years in women and 46.6 (SD 8.3) years in men. Mean height was 161.6 (SD 6.9) cm in women and 172.0 (SD 7.6) cm in men. Overweight or obesity was common in our sample (women: 52%; men: 29%). Overall crude diabetes prevalence was 6.1% (95% CI: 5.0‐7.2).

In Table 1, we show selected characteristics of study participants. Diabetes prevalence was somewhat higher in men than in women (7% and 6%, respectively). In the richest wealth quintile, diabetes prevalence was nearly fourfold higher than in the poorest wealth quintile (11% vs. 3%). Individuals who resided in urban areas were twice as likely to have diabetes compared with those in rural areas (8% vs. 4%). The highest diabetes prevalence occurred in participants with obesity (13%).

**Table 1:** Selected characteristics of study participants

Characteristic	**Total**		**Without diabetes**		**With diabetes**	
	***N***	**Mean or %**	***n***	**Mean or %**	***n***	**Mean or %**
**Height (cm)**	3,241	165.7	3,025	165.7	216	166.3
**Sex**						
**Male**	1,343	39.5%	1,250	93.2%	93	6.8%
**Female**	1,898	60.6%	1,775	94.4%	123	5.6%
**Education**						
**None**	518	14.9%	497	96.1%	21	3.9%
**Primary**	1,083	33.6%	1,019	95.0%	64	5.1%
**Secondary**	1,393	42.8%	1,291	93.3%	102	6.7%
**Higher**	247	8.7%	218	89.3%	29	10.7%
**Wealth index**						
**Poorest**	566	19.6%	549	97.2%	17	2.8%
**Poorer**	595	18.5%	574	97.2%	21	2.8%
**Middle**	638	19.5%	608	95.4%	30	4.6%
**Richer**	755	22.0%	688	91.4%	67	8.6%
**Richest**	687	20.5%	606	89.1%	81	10.9%
**Residency**						
**Urban**	1,510	46.7%	1,375	91.8%	135	8.2%
**Rural**	1,731	53.3%	1,650	95.8%	81	4.2%
**BMI**						
**Thin (<18.5)**	368	11.8%	354	96.5%	14	3.5%
**Normal (18.5‐24.9)**	1,499	47.7%	1,438	96.5%	61	3.5%
**Overweight (25‐29.9)**	722	21.9%	663	92.6%	59	7.4%
**Obesity (>30.0)**	652	18.5%	570	87.2%	82	12.8%

Sample weights used as provided by Namibia Demographic and Health Survey.

In Tables 2, 3, 4, we present our main results. Table 2 shows the pooled data, including both women and men. In Model 1, the odds of having diabetes increased by 1% with every 1‐cm increase in height. This association reversed after adjustment for demographic and socioeconomic variables as well as for BMI in Model 3, although none of the associations in the pooled sample reached statistical significance. The association between height and diabetes differed considerably in sex‐stratified analyses (*P *= 0.003 for height*sex interaction, pooled model). As hypothesized, we observed an inverse relationship among women between height and diabetes in all three models (Table 3). In the unadjusted Model 1, the odds of having diabetes decreased by 3% with every 1‐cm height gain (95% CI: 0.94‐0.99; *P *= 0.033). This relationship persisted after adjustment for demographic and socioeconomic variables as well as for BMI. In Models 2 and 3, the odds of having diabetes among women decreased by 3% and 4% with every 1‐cm increase in height, respectively (95% CI: 0.94‐0.99; *P *= 0.041; and 95% CI: 0.94‐0.99; *P *= 0.023). Interestingly, in contrast to women, diabetes in men showed a positive association with height (Table 4). The odds of having diabetes was 4% higher with every 1‐cm height gain in Model 1 (95% CI: 1.01‐1.08; *P *= 0.021), although this association was somewhat attenuated and not statistically significant after further adjustment for demographic and socioeconomic variables as well as for BMI. Supporting Information Figure S1 displays the discordant relationship between height and FPG values by sex.

**Table 2:** Logistic regression results: association between height and diabetes in Namibia, 2013

**Characteristic**	**Model 1**			**Model 2**			**Model 3**		
	**OR**	**95% CI**	***P***	**OR**	**95% CI**	***P***	**OR**	**95% CI**	***P***
Height (cm)	1.01	0.99‐1.03	0.45	0.99	0.97‐1.02	0.70	0.99	0.97‐1.02	0.47
Age (y)				1.05	1.03‐1.07	<0.001***	1.05	1.02‐1.07	<0.001***
**Sex**									
Male (ref.)				1			1		
Female				0.84	0.60‐1.18	0.38	0.64	0.44‐0.92	0.022**
**Education**									
None (ref.)				1			1		
Primary				1.29	0.65‐2.56	0.47	1.29	0.65‐2.56	0.47
Secondary				1.41	0.72‐2.75	0.30	1.43	0.73‐2.79	0.30
Higher				1.60	0.73‐3.50	0.24	1.51	0.70‐3.27	0.30
**Wealth index**									
Poorest				0.67	0.31‐1.44	0.31	0.81	0.38‐1.75	0.59
Poorer				0.64	0.30‐1.34	0.24	0.69	0.33‐1.48	0.34
Middle (ref.)				1			1		
Richer				1.81	1.04‐3.23	0.037**	1.50	0.87‐2.60	0.15
Richest				2.17	1.17‐4.02	0.014**	1.61	0.86‐3.00	0.13
**Residency**									
Urban (ref.)				1			1		
Rural				0.82	0.50‐1.34	0.42	0.82	0.50‐1.35	0.43
**BMI**									
Thin (<18.5)							1.13	0.58‐2.18	0.72
Normal (18.5‐25) (ref.)							1		
Overweight (25‐29.9)							1.66	1.05‐2.61	0.030**
Obesity (>30.0)							2.98	1.92‐4.63	<0.001***
Observations	3,241			3,241			3,241		

Estimates of odds ratios (ORs) and 95% CIs of explanatory variables for diabetes obtained from logistic regression models. Sample includes women and men aged 35 to 64 included in 2013 Namibia Demographic and Health Survey.

** *P* < 0.05.

*** *P* < 0.01.

Ref., reference category.

**Table 3:** Logistic regression results: association between height and diabetes among women in Namibia, 2013

	**Model 1**			**Model 2**			**Model 3**		
**Characteristic**	**OR**	**95% CI**	***P***	**OR**	**95% CI**	***P***	**OR**	**95% CI**	***P***
**Height (cm)**	0.97	0.94‐0.99	0.033**	0.97	0.94‐0.99	0.041**	0.96	0.94‐0.99	0.023**
**Age (y)**				1.04	1.01‐1.07	0.008***	1.04	1.01‐1.07	0.012**
**Education**									
**None (ref.)**				1			1		
**Primary**				0.84	0.35‐2.01	0.70	0.86	0.36‐2.02	0.72
**Secondary**				0.81	0.32‐2.01	0.65	0.82	0.33‐1.99	0.72
**Higher**				0.69	0.22‐2.19	0.52	0.66	0.21‐2.04	0.47
**Wealth index**									
**Poorest**				0.32	0.11‐0.93	0.037**	0.41	0.14‐1.19	0.10
**Poorer**				0.84	0.37‐1.91	0.67	0.92	0.40‐2.13	0.85
**Middle (ref.)**				1			1		
**Richer**				2.47	1.25‐4.88	0.009***	2.09	1.07‐4.06	0.030**
**Richest**				2.43	1.04‐5.68	0.041**	1.98	0.84‐4.62	0.12
**Residency**									
**Urban (ref.)**				1			1		
**Rural**				0.85	0.45‐1.60	0.62	0.87	0.46‐1.63	0.66
**BMI**									
**Thin (<18.5)**							0.58	0.19‐1.78	0.34
**Normal (18.5‐25) (ref.)**							1		
**Overweight (25‐29.9)**							1.19	0.64‐2.22	0.59
**Obesity (>30.0)**							2.20	1.25‐3.85	0.006***
**Observations**	1,898			1,898			1,898		

Estimates of odds ratios (ORs) and 95% CIs of explanatory variables for diabetes obtained from logistic regression models. Sample includes women ages 35 to 64 included in 2013 Namibia Demographic and Health Survey.

** *P* < 0.05.

*** *P* < 0.01.

Ref., reference category.

**Table 4:** Logistic regression results: association between height and diabetes among men in Namibia, 2013

**Characteristic**	**Model 1**			**Model 2**			**Model 3**		
	**OR**	**95% CI**	***P***	**OR**	**95% CI**	***P***	**OR**	**95% CI**	***P***
**Height (cm)**	1.04	1.01‐1.08	0.021**	1.02	0.99‐1.06	0.17	1.02	0.98‐1.05	0.38
**Age (y)**				1.06	1.03‐1.09	<0.001***	1.05	1.02‐1.09	0.002***
**Education**									
None (ref.)				1			1		
Primary				2.40	0.68‐8.52	0.18	2.37	0.66‐8.51	0.18
Secondary				3.64	0.96‐13.77	0.057*	3.27	0.85‐12.57	0.084*
Higher				4.96	1.25‐19.77	0.023**	4.36	1.09‐17.45	0.038**
**Wealth index**									
Poorest				1.47	0.50‐4.30	0.48	1.50	0.50‐4.48	0.47
Poorer				0.28	0.07‐1.09	0.066*	0.28	0.07‐1.11	0.071*
Middle (ref.)				1			1		
Richer				1.14	0.50‐2.63	0.76	0.93	0.42‐2.06	0.85
Richest				1.70	0.76‐3.83	0.20	1.14	0.52‐2.49	0.74
**Residency**									
Urban (ref.)				1			1		
Rural				0.84	0.45‐1.58	0.59	0.83	0.44‐1.56	0.56
**BMI**									
Thin (<18.5)							1.60	0.70‐3.67	0.26
Normal (18.5‐25) (ref.)							1		
Overweight (25‐29.9)							2.08	1.07‐4.03	0.030**
Obesity (>30.0)							3.57	1.95‐6.53	<0.001***
Observations	1,343			1,343			1,343		

Estimates of odds ratios (ORs) and 95% CIs of explanatory variables for diabetes obtained from logistic regression models. Sample includes men aged 35 to 64 included in 2013 Namibia Demographic and Health Survey.

* *P* < 0.1.

** *P* < 0.05.

*** *P* < 0.01.

Ref., reference category.

Obesity was associated with a substantial increase in the odds of having diabetes (220% in women [95% CI: 1.25‐3.85; *P *= 0.006] and 357% in men [95% CI: 1.95‐6.53; *P *< 0.001]). Supporting Information Figure S2 shows the distribution of BMI in each height quartile in individuals with diabetes, separately by sex. In women with diabetes, obesity was inversely distributed over height quartiles and occurred most frequently in the shortest height category. Based on this observation, a mediating role of BMI in the development of diabetes appears likely. However, after full adjustment including BMI in Model 3, the protective relationship between height and diabetes persisted, suggesting that height in women was independently related to diabetes. In contrast, the highest prevalence of obesity in men with diabetes occurred in the tallest height category.

Our results were consistent across a wide range of robustness checks (Supporting Information Tables S1‐S10). Using height quartiles instead of height as a continuous variable, the inverse relationship in women was consistent across all three models. In Model 3, for women in the tallest height quartile (Q4), the odds of having diabetes was 43% smaller compared with those in the shortest height quartile (Q1) (*P *= 0.082); however, in men, no relationship was found. Including a polynomial for age in our models, the odds of having diabetes decreased by 4% with every 1‐cm height gain (95% CI: 0.94‐0.99; *P *= 0.030; Model 3; female sample). Supporting Information Figure S3 shows the relationship between the proportion of early childhood stunting and mean attained adult height by region in Namibia. As expected, attained adult height was highly correlated with the proportion of children under age 5 years who were stunted (*P *< 0.001). In the placebo test, limiting our sample to wife/husband dyads, the protective relationship between height and diabetes was observed only for wife’s height and wife’s diabetes status.

## Discussion

Using nationally representative data from Namibia, we found that height was related to reduced odds of having diabetes in women but not in men. The effect in women was robust to adjustment for demographic and socioeconomic variables, as well as BMI, suggesting an independent effect of height on diabetes. In contrast, we found a positive association between height and diabetes in men, but this effect was attenuated and not significant after full adjustment for covariates, suggesting possible confounding by covariates. These findings suggest not only sex‐specific differences in the direction of the relationship between height and diabetes risk but also in the role of covariates. Our findings were consistent across a range of robustness checks, including alternative specifications of our exposure. To our knowledge, this analysis is the first to examine the relationship between height and diabetes using a nationally representative survey design in Africa. It therefore fills a major knowledge gap in the evaluation of this relationship in a context where stunting is highly prevalent.

Our findings are supported by a meta‐analysis of nine studies that demonstrated a similar inverse relationship between height and diabetes in women but not in men 26. However, most prior studies have been carried out in upper‐middle and high‐income countries where early childhood malnutrition is relatively uncommon. In contrast, Namibia has one of the highest levels of early childhood stunting globally, with 21% and 27% of female and male children under age 5 years being stunted in 2013 27. A study in a Nigerian urban population reported that height was associated with abnormal glucose tolerance (diabetes and impaired glucose tolerance) but without further control for potential confounders 36. Two studies from Bangladesh used nationally representative data from the DHS and found an inverse association between height and diabetes but without sex differentiation 37, 38.

Though growing evidence has pointed to height as an independent risk factor of impaired glucose metabolism and development of diabetes, the mechanisms remain unclear. Although a few studies have been conducted in humans, animal models have demonstrated structural and functional changes in tissues and organs involved in glucose metabolism after nutritional restriction during organogenesis or early in life. Other hypothesized pathways include epigenetic modifications, leading to permanent changes in gene expression, and mitochondrial dysfunction, leading to cumulative oxidative damage 6. However, only little is known about sex‐specific differences in these pathways that may lead to an unequal outcome despite the shared exposition of an adverse environment in early life. Furthermore, the potential mediating role of BMI on disease development should be considered, particularly the time when weight gain occurs. In girls, rapid weight gain in prepubertal years would lead to an earlier onset of puberty and, consequently, to faster bone maturation and reduced final height; however, in boys, obesity in childhood would be associated with delayed puberty and taller height 39, 40. Future research should investigate sex differences, including possible sex‐specific compensating mechanisms after exposure to malnutrition in pregnancy or early childhood.

A potential causal link between height and diabetes in women would have wide‐ranging policy implications. Food security is a priority for regional and international policy makers, particularly with the institution of the Sustainable Development Goals. Sustainable Development Goal number 2 focuses on “achieving food security and improving nutrition,” with a special focus on the first 1,000 days of life. Estimates of the returns to investments in improving early life living standards would be inaccurate without taking into account the full array of long‐run health benefits 4, 18. This would be highly relevant for the African context, where the agricultural sector is deeply interwoven with the health sector, and subsistence farming and herding are common. Other policy implications could include changes in public health programming targeted to shorter women, such as enhanced diabetes screening.

This study has several limitations. First, causality cannot be determined with the current study design. The associations described here should generate hypotheses for further studies, including multicountry and (quasi‐)experimental research to assess causal effects and pathways in settings where stunting is prevalent. Quasi‐experimental studies, for instance, could exploit birth cohort‐ or region‐specific exposure to famines to identify differential exposure to malnourishment in childhood 41. Second, we were limited by the data at hand. We did not have a direct metric of early childhood malnutrition and used attained adult height as a proxy for nutritional status in childhood. With this approach, a participant with exposure to childhood stunting and later catch‐up growth would not be differentiated from a well‐fed person with normal growth, which may have led to misclassification of our exposure status. Adults may also be of shorter height for a range of other reasons that are unrelated to early childhood living conditions, including genetic, metabolic, and endocrine factors 42. Nevertheless, stunted children have only a small window of opportunity for catch‐up growth 4, 12, and attained adult height has been shown to be a measure of cumulative net nutrition on a population level 32. We also did not have information on several behavioral factors that may contribute to the pathogenesis of diabetes, such as soft drink consumption and exercise. Third, using FPG alone fails to diagnose about 30% of cases of previously undiagnosed diabetes 43. Similarly, although the prevalence of type 1 diabetes is low in sub‐Saharan Africa, we were unable to differentiate between types of diabetes. Gestational diabetes was low in our sample (one respondent). Fourth, although respondents confirmed having fasted prior to the FPG test, this may not have been the case because of social desirability bias. Nevertheless, overreporting of adherence to fasting is unlikely to affect our results so long as it is not related to a respondent’s height, net of the covariates included in our models. Fifth, our findings may not generalize beyond Namibia, and the mechanisms underlying the relationship between height and diabetes may vary across contexts 6. These mechanisms may also be independent from adiposity 44. Adiposity does not appear to be a prerequisite for adult diabetes in shorter individuals, but rather relative weight gain compared with the individual growth trajectory appears to influence disease development 4.

## Conclusion

Shorter height is associated with increased diabetes in women but not in men in Namibia. Exposure to adverse environments in early life may lead to impaired glucose metabolism and diabetes later in life in this subpopulation. If further research confirms our findings, then implementing interventions that reduce childhood stunting and allow women to reach their full growth potential might help prevent the growing diabetes burden in the region.

## References

[1] World Health Organization . Global Report on Diabetes. Geneva: WHO; 2016.

[2] Manne‐Goehler J , Atun R , Stokes A , et al. Diabetes diagnosis and care in sub‐Saharan Africa: pooled analysis of individual data from 12 countries. Lancet Diabetes Endocrinol 2016;4:903‐912. 27727123 10.1016/S2213-8587(16)30181-4

[3] Tuei VC , Maiyoh GK , Ha CE . Type 2 diabetes mellitus and obesity in sub‐Saharan Africa. Diabetes Metab Res Rev 2010;26:433‐445. 20641142 10.1002/dmrr.1106

[4] Victora CG , Adair L , Fall C , et al. Maternal and child undernutrition: consequences for adult health and human capital. Lancet 2008;371:340‐357. 18206223 10.1016/S0140-6736(07)61692-4 PMC2258311

[5] Hales CN , Barker DJP . Type 2 (non‐insulin‐dependent) diabetes mellitus: the thrifty phenotype hypothesis. Diabetologia 1992;35:595‐601. 1644236 10.1007/BF00400248

[6] Warner MJ , Ozanne SE . Mechanisms involved in the developmental programming of adulthood disease. Biochem J 2010;427:333‐347. 20388123 10.1042/BJ20091861

[7] Langley‐Evans SC . Nutrition in early life and the programming of adult disease: a review. J Hum Nutr Diet 2015;28(suppl 1):1‐14. 10.1111/jhn.12212 24479490

[8] Prentice AM , Moore SE . Early programming of adult diseases in resource poor countries. Arch Dis Child 2005;90:429‐432. 15781942 10.1136/adc.2004.059030 PMC1720333

[9] Godfrey KM , Inskip HM , Hanson MA . The long‐term effects of prenatal development on growth and metabolism. Semin Reprod Med 2011;29:257‐265. 21769765 10.1055/s-0031-1275518 PMC3685133

[10] World Health Organization . Nutrition Landscape Information System (NLIS) Country Profile Indicators: Interpretation Guide. Geneva: WHO; 2010.

[11] Prendergast AJ , Humphrey JH . The stunting syndrome in developing countries. Paediatr Int Child Health 2014;34:250‐265. 25310000 10.1179/2046905514Y.0000000158 PMC4232245

[12] de Onis M , Branca F . Childhood stunting: a global perspective. Matern Child Nutr 2016;12(suppl 1):12‐26. 27187907 10.1111/mcn.12231 PMC5084763

[13] Caulfield LE , Richard SA , Rivera JA , Musgrove P , Black RE . Stunting, wasting, and micronutrient deficiency disorders In: Jamison DT, Breman JG, Measham AR, et al., eds. Disease Control Priorities in Developing Countries. 2nd ed. Washington, DC: The International Bank for Reconstruction and Development/The World Bank Group; New York: Oxford University Press; 2006. 21250337

[14] Stein AD , Wang M , Martorell R , et al. Growth patterns in early childhood and final attained stature: data from five birth cohorts from low‐ and middle‐income countries. Am J Hum Biol 2010;22:353‐359. 19856426 10.1002/ajhb.20998 PMC3494846

[15] Victora CG , de Onis M , Hallal PC , Blossner M , Shrimpton R . Worldwide timing of growth faltering: revisiting implications for interventions. Pediatrics 2010;125:e473‐e480. 20156903 10.1542/peds.2009-1519

[16] Ozaltin E , Hill K , Subramanian SV . Association of maternal stature with offspring mortality, underweight, and stunting in low‐ to middle‐income countries. JAMA 2010;303:1507‐1516. 20407060 10.1001/jama.2010.450 PMC3100588

[17] Whincup PH , Kaye SJ , Owen CG , et al. Birth weight and risk of type 2 diabetes: a systematic review. JAMA 2008;300:2886‐2897. 19109117 10.1001/jama.2008.886

[18] Adair LS , Fall CHD , Osmond C , et al. Associations of linear growth and relative weight gain during early life with adult health and human capital in countries of low and middle income: findings from five birth cohort studies. Lancet 2013;382:525‐534. 23541370 10.1016/S0140-6736(13)60103-8 PMC3744751

[19] Janghorbani M , Amini M . Associations of hip circumference and height with incidence of type 2 diabetes: the Isfahan diabetes prevention study. Acta Diabetol 2012;49(suppl 1):S107‐S114. 22080142 10.1007/s00592-011-0351-4

[20] Baker JL , Furer A , Afek A , et al. Height at late adolescence and incident diabetes among young men. PLoS One 2015;10:e0136464. doi:10.1371/journal.pone.0136464 26305680 PMC4549289

[21] Bjerregaard LG , Jensen BW , Baker JL . Height at ages 7–13 years in relation to developing type 2 diabetes throughout adult life. Paediatr Perinat Epidemiol 2017;31:284‐292. 28590597 10.1111/ppe.12366

[22] Han TS , Feskens EJM , Lean MEJ , Seidell JC . Associations of body composition with type 2 diabetes mellitus. Diabet Med 1998;15:129‐135. 9507913 10.1002/(SICI)1096-9136(199802)15:2<129::AID-DIA535>3.0.CO;2-2

[23] Asao K , Kao WHL , Baptiste‐Roberts K , Bandeen‐Roche K , Erlinger TP , Brancati FL . Short stature and the risk of adiposity, insulin resistance, and type 2 diabetes in middle age: the Third National Health and Nutrition Examination Survey (NHANES III), 1988‐1994 *. * Diabetes Care 2006;29:1632‐1637. 16801590 10.2337/dc05-1997

[24] Perelman J . Are chronic diseases related to height? Results from the Portuguese National Health Interview Survey. Econ Hum Biol 2014;15:56‐66. 25062533 10.1016/j.ehb.2014.06.001

[25] Schulze MB , Heidemann C , Schienkiewitz A , Bergmann MM , Hoffmann K , Boeing H . Comparison of anthropometric characteristics in predicting the incidence of type 2 diabetes in the EPIC‐potsdam study. Diabetes Care 2006;29:1921‐1923. 16873804 10.2337/dc06-0895

[26] Janghorbani M , Momeni F , Dehghani M . Hip circumference, height and risk of type 2 diabetes: systematic review and meta‐analysis. Obes Rev 2012;13:1172‐1181. 22943765 10.1111/j.1467-789X.2012.01030.x

[27] The Nambia Ministry of Health and Social Services (MoHSS), ICF International . The Namibia Demographic and Health Survey 2013. Windhoek, Namibia, and Rockville, MD: MoHSS and ICF International; 2014.

[28] Mtambo OPL , Katoma V , Kazembe LNM . Analysis of severe childhood stunting in Namibia. Int J Stat Appl 2016;6:81‐88.

[29] NCD Risk Factor Collaboration . Trends in adult body‐mass index in 200 countries from 1975 to 2014: a pooled analysis of 1698 population‐based measurement studies with 19·2 million participants. Lancet 2016;387:1377‐1396. 27115820 10.1016/S0140-6736(16)30054-X PMC7615134

[30] NCD Risk Factor Collaboration. Evolution of diabetes over time 2017. http://ncdrisc.org/diabetes-prevalence-line.html. Published 2017. Accessed July 15, 2018.

[31] Karlsson O , De Neve J‐W , Subramanian SV . Weakening association of parental education: analysis of child health outcomes in 43 low‐ and middle‐income countries [published online August 7, 2018]. Int J Epidemiol 2018. doi:10.1093/ije/dyy158 30101357

[32] Perkins JM , Subramanian SV , Davey Smith G , Ozaltin E . Adult height, nutrition, and population health. Nutr Rev 2016;74:149‐165. 26928678 10.1093/nutrit/nuv105 PMC4892290

[33] World Health Organization . Definition and Diagnosis of Diabetes Mellitus and Intermediate Hyperglycemia: Report of a WHO/IDF Consultation. Geneva: WHO; 2006.

[34] World Health Organization . Obesity Preventing and Managing the Global Epidemic of a WHO Consultation. WHO Technical Report Series no. 894. Geneva: WHO; 2000. 11234459

[35] Epstein E , Guttman R . Mate selection in man: evidence, theory, and outcome. Soc Biol 1984;31:243‐278. 6400148 10.1080/19485565.1984.9988579

[36] Olatunbosun ST , Bella AF . Relationship between height, glucose tolerance, and hypertension in an urban African black adult population: a case for the "Thrifty Phenotype Hypothesis"? J Natl Med Assoc 2000;92:265‐268. 10918760 PMC2640520

[37] Hoque ME , Khokan MR , Bari W . Impact of stature on non‐communicable diseases: evidence based on Bangladesh Demographic and Health Survey, 2011 data. BMC Public Health 2014;14:1007. doi:10.1186/1471-2458-14-1007 25261299 PMC4195861

[38] Talukder A , Mallick TS . On association between diabetes status and stature of individual in Bangladesh: an ordinal regression analysis. Int J Diabetes Dev Ctries 2017;37:470‐477.

[39] Marcovecchio ML , Chiarelli F . Obesity and growth during childhood and puberty. World Rev Nutr Diet 2013;106:135‐141. 23428692 10.1159/000342545

[40] Prentice P , Viner RM . Pubertal timing and adult obesity and cardiometabolic risk in women and men: a systematic review and meta‐analysis. Int J Obes 2013;37:1036‐1043. 10.1038/ijo.2012.177 23164700

[41] De Neve J‐W , Subramanian SV . Causal effect of parental schooling on early childhood undernutrition—quasi‐experimental evidence from Zimbabwe. Am J Epidemiol 2018;187:82‐93. 29309520 10.1093/aje/kwx195

[42] Magiakou MA , Mastorakos G , Oldfield EH , et al. Cushing's syndrome in children and adolescents. Presentation, diagnosis, and therapy. N Engl J Med 1994;331:629‐636. 8052272 10.1056/NEJM199409083311002

[43] Expert Committee on the Diagnosis and Classification of Diabetes Mellitus . Report of the Expert Committee on the Diagnosis and Classification of Diabetes Mellitus. Diabetes Care 2003;26(suppl 1):S5‐S20. 12502614 10.2337/diacare.26.2007.s5

[44] Walker SP , Chang SM , Powell CA . The association between early childhood stunting and weight status in late adolescence. Int J Obes 2007;31:347‐352. 10.1038/sj.ijo.0803383 16718285

