# Supplementary material for: Shorter Height is Associated with Diabetes in Women but not in Men: Nationally Representative Evidence from Namibia
Source: Obesity (Silver Spring). 2019 Feb 25;27(3):505–12. doi: 10.1002/oby.22394 (PMC6646871; doi:10.1002/oby.22394)
Supplement: Supplementary file 1 [file OBY-27-505-s001.docx]

**Appendix to “Shorter height is associated with diabetes in women but not in men: nationally representative evidence from Namibia”**

**Authors:** Viola Koncz, M.D. M.P.H.^1,2^, Pascal Geldsetzer, M.D. Sc.D.^3^, Jennifer Manne-Goehler M.D. Sc.D.^4^, Amanda S. Wendt Ph.D. M.S.^1^, Felix Teufel B.M. ^1^, S. V. Subramanian, Ph.D.^5,6^, Till Bärnighausen, M.D. Sc.D.^1,3,7^, Jan-Walter De Neve, M.D. Sc.D.^1*^

**Affiliations:**

^1^ Institute of Global Health, Medical Faculty and University Hospital, Heidelberg University, Im Neuenheimer Feld 130.3, 69120 Heidelberg, Germany.

^2^ IBE - Institute for Medical Information Processing, Biometry and Epidemiology, Ludwig Maximilian University of Munich, Marchioninistr. 15, 81377 Munich, Germany.

^3^ Department of Global Health and Population, Harvard T.H. Chan School of Public Health, 665 Huntington Avenue, Boston MA 02115, United States of America.

^4^ Department of Medicine, Beth Israel Deaconess Medical Center, Harvard Medical School, 330 Brookline Avenue, Boston, MA 02215, United States of America.

^5^ Department of Social and Behavioral Sciences, Harvard T.H. Chan School of Public Health, 665 Huntington Avenue, Boston MA 02115, United States of America.

^6^ Center for Population and Development Studies, Harvard T.H. Chan School of Public Health, 9 Bow Street, Cambridge MA 02138, United States of America.

^7^ Africa Health Research Institute, Mtubatuba 3935, KwaZulu-Natal, South Africa.

***Correspondence:** Institute of Global Health, Medical Faculty and University Hospital, Heidelberg University, Im Neuenheimer Feld 130.3, R.314, 69120 Heidelberg, Germany. E-mail: [janwalter.deneve@uni-heidelberg.de](mailto:janwalter.deneve@uni-heidelberg.de). Phone: +49-6221-5632873.

In this supplementary appendix, we provide additional details related to our study, including:

- **Figure S1**. Fasting plasma glucose values by height (continuous) and sex in Namibia
- **Figure S2**. BMI categories per height quartile in individuals with diabetes in Namibia
- **Figure S3**. Early childhood stunting and attained adult height by region in Namibia
- **Table S1**. Association between height quartiles and diabetes among women in Namibia
- **Table S2**. Association between height quartiles and diabetes among men in Namibia
- **Table S3**. Association between height and diabetes, using quadratic and cubic terms in age
- **Table S4**. Association between height and diabetes, using quadratic and cubic terms in BMI
- **Table S5**. Association between height and diabetes, using BMI as a continuous variable
- **Table S6**: Association between height and diabetes, controlling for occupation in workers
- **Table S7**. Association between height and diabetes, using Poisson log link function
- **Table S8**. Association between height and diabetes, excluding pregnant women
- **Table S9**. Placebo test using subsample of wife/husband dyads, wife’s diabetes status
- **Table S10**. Placebo test using subsample of wife/husband dyads, husband’s diabetes status

Figure S1. Height and fasting plasma glucose in Namibia, by sex


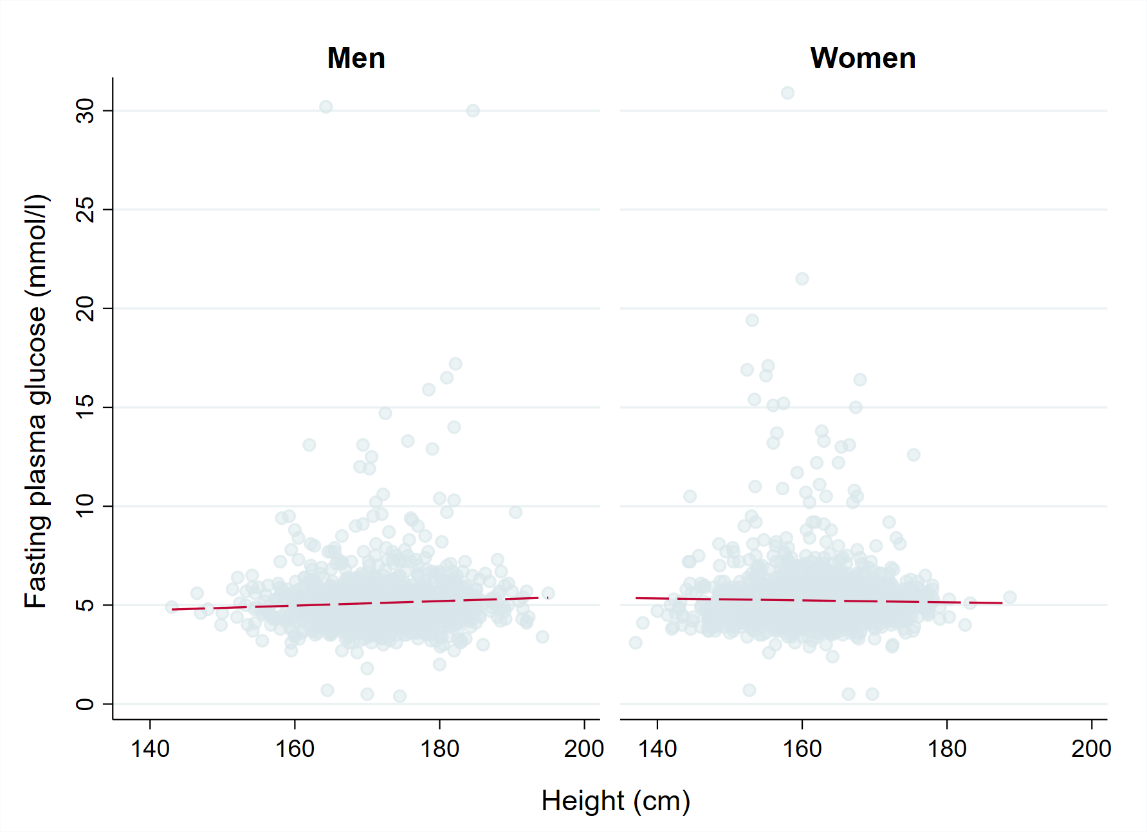


*Notes:* Figure S1 shows the relationship between height and fasting plasma glucose in Namibia by sex in 2013. The line represents unadjusted linear regression showing height weakly positively associated with lower fasting plasma glucose in women; whereas the reverse is observed in men. Circles represent raw data. Source: author’s calculations using data from the Namibia Demographic and Health Survey in 2013.

Figure S2. Distribution of BMI categories per height quartile in individuals with diabetes


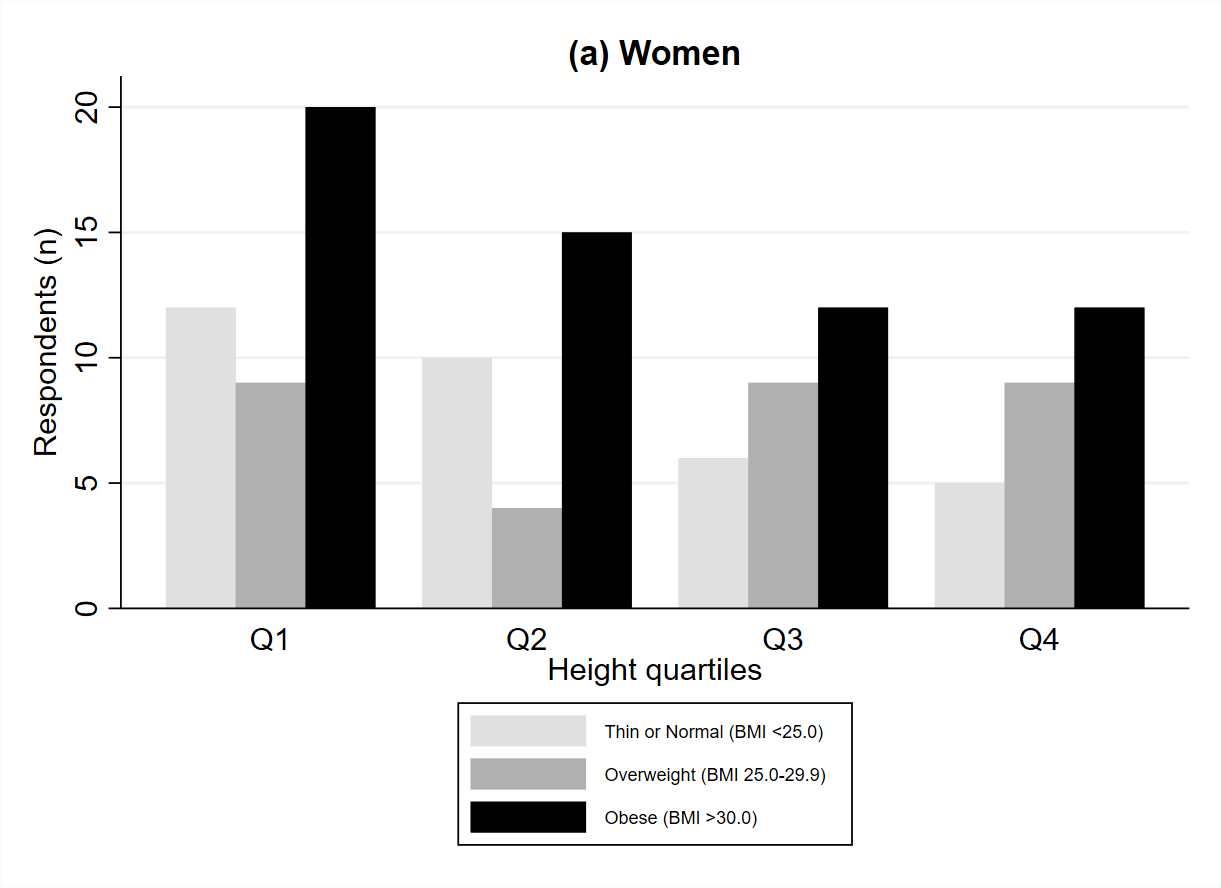


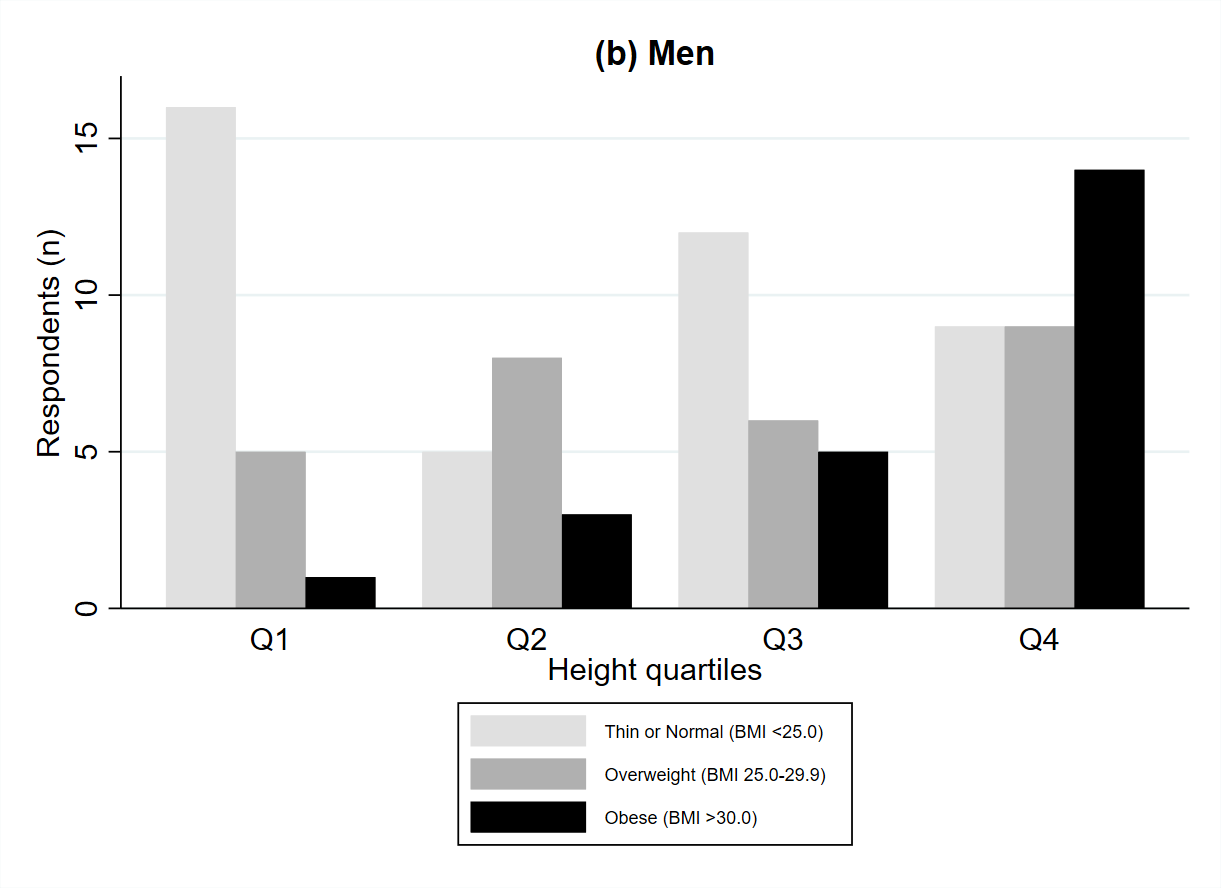


*Notes:* Figure S2 shows BMI categories per height quartile in individuals with diabetes in Namibia, separately for women (a) and men (b). There were more women with diabetes in lower height quartiles with obesity, whereas, conversely, there were more men with diabetes in higher height quartiles with obesity. These results are consistent with those of higher odds of having diabetes among taller women in the main text. Source: author’s calculations using the Namibia Demographic and Health Survey in 2013.

Figure S3. Early childhood stunting and attained adult height by region in Namibia


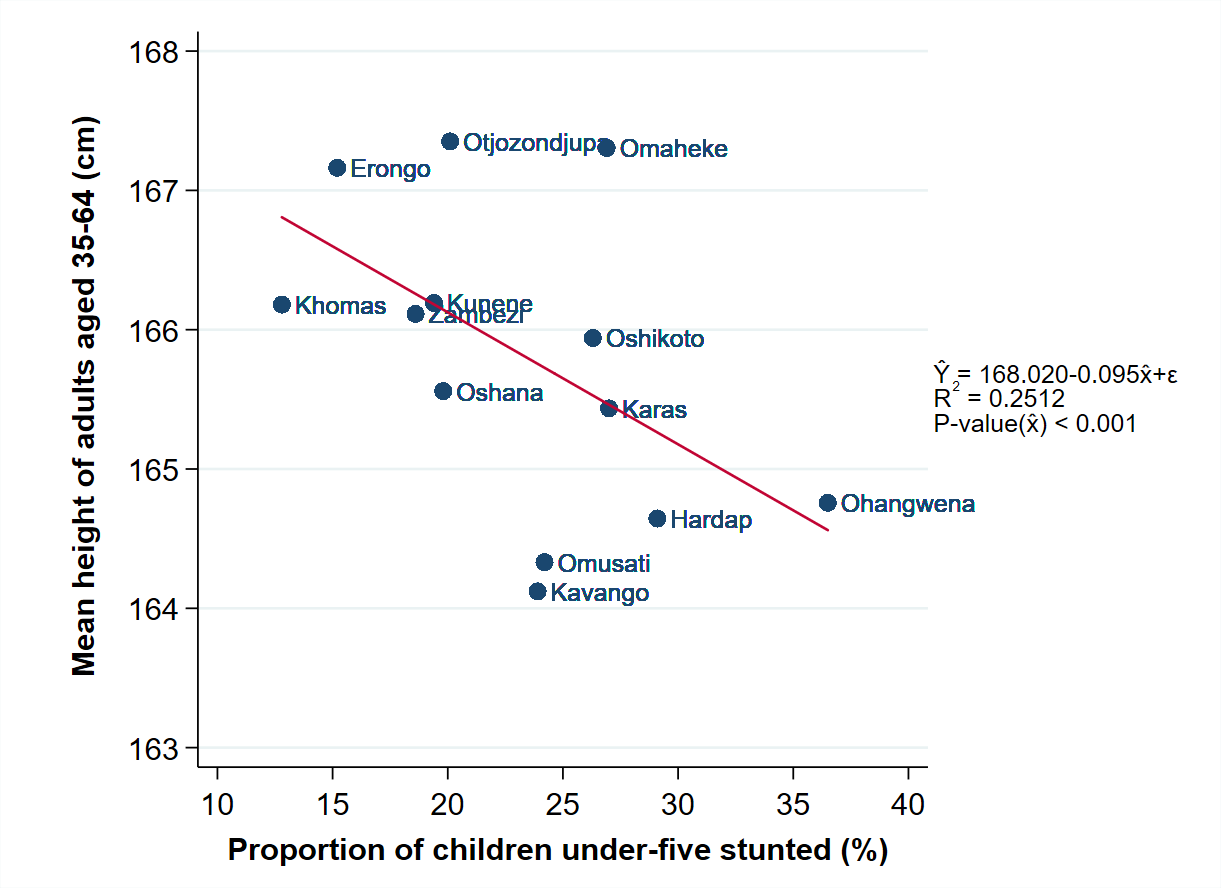


*Notes:* Figure S3 shows the relationship between the proportion of children under-five that are stunted (%) and the mean attained height of adults in our sample (cm) by region in Namibia. Stunting was defined as defined as below −2 standard deviations from the median of the WHO 2006 reference population in terms of height-for-age.

Table S1. Logistic regression results: the association between height quartiles and diabetes among women in Namibia

*Notes:* Estimates of odds ratios (OR) and 95% confidence intervals (CI) of explanatory variables for diabetes obtained from logistic regression models. Sample includes women ages 35 - 64 included in the Demographic and Health Survey in 2013. *** p<0.01, ** p<0.05, * p<0.1. Abbreviations: BMI: Body Mass Index. Ref: reference category.

Table S2. Logistic regression results: the association between height quartiles and diabetes among men in Namibia

*Notes:* Estimates of odds ratios (OR) and 95% confidence intervals (CI) of explanatory variables for diabetes obtained from logistic regression models. Sample includes men ages 35 - 64 included in the Demographic and Health Survey in 2013. *** p<0.01, ** p<0.05, * p<0.1. Abbreviations: BMI: Body Mass Index. Ref: reference category.

Table S3. Association between height and diabetes, using quadratic and cubic terms in age

*Notes:* Estimates of odds ratios (OR) and 95% confidence intervals (CI) of explanatory variables for diabetes obtained from logistic regression models. Sample includes respondents ages 35 - 64 included in the Demographic and Health Survey in 2013. BMI was included as four categories, including thin (BMI < 18.5), normal (BMI 18.5-24.9), overweight (BMI 25-29.9) and obesity (BMI ≥ 30). *** p<0.01, ** p<0.05, * p<0.1. Abbreviations: BMI: Body Mass Index.

Table S4. Association between height and diabetes, using quadratic and cubic terms in Body Mass Index

*Notes:* Estimates of odds ratios (OR) and 95% confidence intervals (CI) of explanatory variables for diabetes obtained from logistic regression models. Sample includes respondents ages 35 - 64 included in the Demographic and Health Survey in 2013. *** p<0.01, ** p<0.05, * p<0.1. BMI was a continuous variable. Abbreviations: BMI: Body Mass Index.

Table S5. Association between height and diabetes, using Body Mass Index as a continuous variable

*Notes:* Estimates of odds ratios (OR) and 95% confidence intervals (CI) of explanatory variables for diabetes obtained from logistic regression models. Sample includes respondents ages 35 - 64 included in the Demographic and Health Survey in 2013. BMI was included as a continuous variable. *** p<0.01, ** p<0.05, * p<0.1. Abbreviations: BMI: Body Mass Index.

Table S6. Association between height and diabetes, controlling for occupation in workers

*Notes:* Estimates of odds ratios (OR) and 95% confidence intervals (CI) of explanatory variables for diabetes obtained from logistic regression models. Sample includes respondents ages 35 - 64 included in the Demographic and Health Survey 2013. BMI was included as four categories, including thin (BMI < 18.5), normal (BMI 18.5-24.9), overweight (BMI 25-29.9) and obesity (BMI ≥ 30). Occupation was included as nine categories: managers; professionals; technicians and associate professionals; clerks; service workers and shop and market sales workers; skilled agricultural and fishery workers; craft and related trades workers; plant and machine operators and assemblers; and, finally, elementary occupations (e.g., agricultural laborers). *** p<0.01, ** p<0.05, * p<0.1.

Table S7. Association between height and diabetes, using Poisson log link function

*Notes*: Estimates of relative risks (RR) and 95% confidence intervals (CI) of explanatory variables for diabetes obtained from poisson regression models. Sample includes respondents ages 35 - 64 included in the Demographic and Health Survey 2013. BMI was included as four categories, including thin (BMI < 18.5), normal (BMI 18.5-24.9), overweight (BMI 25-29.9) and obesity (BMI ≥ 30). *** p<0.01, ** p<0.05, * p<0.1. Abbreviations: BMI: Body Mass Index. Ref: reference category.

Table S8. Association between height and diabetes, excluding pregnant women

*Notes:* Estimates of odds ratios (OR) and 95% confidence intervals (CI) of explanatory variables for diabetes obtained from logistic regression models. Sample includes women ages 35 - 64 included in the Demographic and Health Survey in 2013. Women who reported being pregnant were excluded from the analysis (n=35). *** p<0.01, ** p<0.05, * p<0.1. Abbreviations: BMI: Body Mass Index. Ref: reference category.

Table S9. Placebo test using subsample of wife/husband dyads, wife’s diabetes status

*Notes*: Estimates of odds ratios (OR) and 95% confidence intervals (CI) of explanatory variables for diabetes obtained from logistic regression models. Sample includes women ages 35 - 64 who had one partner and were married in the Demographic and Health Survey 2013. BMI was included as four categories, including thin (BMI < 18.5), normal (BMI 18.5-24.9), overweight (BMI 25-29.9) and obesity (BMI ≥ 30). *** p<0.01, ** p<0.05, * p<0.1. Abbreviations: BMI: Body Mass Index.

Table S10. Placebo test using subsample of wife/husband dyads, husband’s diabetes status

*Notes*: Estimates of odds ratios (OR) and 95% confidence intervals (CI) of explanatory variables for diabetes obtained from logistic regression models. Sample includes men ages 35 - 64 who had one partner and were married in the Demographic and Health Survey 2013. BMI was included as four categories, including thin (BMI < 18.5), normal (BMI 18.5-24.9), overweight (BMI 25-29.9) and obesity (BMI ≥ 30). *** p<0.01, ** p<0.05, * p<0.1. Abbreviations: BMI: Body Mass Index. Ref: reference category.
